# Pregnancy Epigenetic Signature in T Helper 17 and T Regulatory Cells in Multiple Sclerosis

**DOI:** 10.3389/fimmu.2018.03075

**Published:** 2019-01-08

**Authors:** Andrea Iannello, Simona Rolla, Alessandro Maglione, Giulio Ferrero, Valentina Bardina, Ilenia Inaudi, Stefania De Mercanti, Francesco Novelli, Lucrezia D'Antuono, Simona Cardaropoli, Tullia Todros, Maria Vittoria Turrini, Cinzia Cordioli, Giorgia Puorro, Angela Marsili, Roberta Lanzillo, Vincenzo Brescia Morra, Francesca Cordero, Michele De Bortoli, Luca Durelli, Andrea Visconti, Santina Cutrupi, Marinella Clerico

**Affiliations:** ^1^Department of Clinical and Biological Sciences, University of Turin, Turin, Italy; ^2^Department of Computer Science, University of Turin, Turin, Italy; ^3^Department of Molecular Biotechnology and Healthy Sciences, University of Turin, Turin, Italy; ^4^Obstetric and Gynecologic Department, OIRM-Sant'Anna Hospital, Città della Salute e della Scienza, Turin, Italy; ^5^Department of Surgical Sciences, University of Turin, Turin, Italy; ^6^Multiple Sclerosis Center, Ospedali Civili di Brescia, Montichiari Hospital, Montichiari, Italy; ^7^Department of Neurosciences, Reproductive and Odontostomatological Sciences, University of Naples Federico II, Naples, Italy; ^8^Medical Affairs Department, Merck Serono S.p.A, Rome, Italy

**Keywords:** pregnancy, epigenetic profile, RORC, FOXP3, Th17, Treg, multiple sclerosis, ERα

## Abstract

Increasing evidence supports the anti-inflammatory role of estrogens in Multiple Sclerosis (MS), originating from the observation of reduction in relapse rates among women with MS during pregnancy, but the molecular mechanisms are still not completely understood. Using an integrative data analysis, we identified T helper (Th) 17 and T regulatory (Treg) cell-type-specific regulatory regions (CSR) regulated by estrogen receptor alpha (ERα). These CSRs were validated in polarized Th17 from healthy donors (HD) and in peripheral blood mononuclear cells, Th17 and Treg cells from relapsing remitting (RR) MS patients and HD during pregnancy. 17β-estradiol induces active histone marks enrichment at Forkhead Box P3 (FOXP3)-CSRs and repressive histone marks enrichment at RAR related orphan receptor C (RORC)-CSRs in polarized Th17 cells. A disease-associated epigenetic profile was found in RRMS patients during pregnancy, suggesting a FOXP3 positive regulation and a RORC negative regulation in the third trimester of pregnancy. Altogether, these data indicate that estrogens act as immunomodulatory factors on the epigenomes of CD4+ T cells in RRMS; the identified CSRs may represent potential biomarkers for monitoring disease progression or new potential therapeutic targets.

## Introduction

Multiple Sclerosis (MS) is an autoimmune disease characterized by chronic inflammation of the central nervous system (CNS) affecting 2.5 million people worldwide, with a female/male sex ratio of 3:1 (1, 2). Pro-inflammatory T helper (Th) 17 cells are required for the pathogenesis of MS (3, 4) and its mouse model, the experimental autoimmune encephalomyelitis (EAE), whereas CD4+Foxp3+ regulatory T cells (Treg), crucial for preventing autoimmunity, are defective in numbers and functions (5). Intriguingly, the female sex hormone estrogen is protective in MS: it exerts potent effects on immune cells and in the CNS during pregnancy, especially in the third trimester when they peak and the most pronounced decrease in the relapse rate occurs (6). This potent, short-term beneficial effect of pregnancy is then followed by a temporary rebound of disease activity post-partum, probably due to the fall of estrogen serum concentration (7).

The role of estrogen-induced immunomodulation is well-demonstrated both on innate immune cells and on adaptive immune cells (8), however little is known about the molecular mechanism underlying its action on the immune system. Estrogens act by binding Estrogen Receptors (ER) α and β that, functioning as ligand-activated transcription factors, bind specific DNA sequences, associate chromatin remodelers and transcriptional factors, and therefore regulate a broad range of estrogen-responsive genes. Among T lymphocytes, CD4+ T cells express higher levels of ERα than ERβ (9) and ERα signaling is required for estrogen-mediated regulation of CD4+ T cell subsets and protection against EAE (10, 11). In the EAE model, estrogens have been shown to have an anti-inflammatory effect by inhibiting CD4+ T cells expansion, decreasing autoantigen-specific Th1 and Th17 cells (12, 13) and increasing proportion of Treg cells (14, 15). In MS patients, the protective effect of estrogens has been reported in a pivotal trial (16, 17) and currently, large placebo-controlled clinical trials of estrogen therapy in MS are still ongoing (18).

CD4+ T cells, after being activated, differentiate into distinct effector subsets, characterized by the expression of specific Transcription Factors (TF), cytokines, cytokine receptors, and surface molecules that drive different immunomodulatory features (19). Each cell type has its own unique chromatin landscape that defines cell identity and its specific functions. However, these cells retain the ability to change their identity and adapt their functions upon new polarizing environments that act on cell-type specific epigenetic features. Interestingly, the balance between Th17 and Treg cells, that have a central role in MS outcome (20), depends on epigenetic dynamics (21). These pivotal regulatory nodes can divert T cell functions toward inflammatory or regulatory state reprogramming T cells and modulating immune response (22, 23).

Epigenomic profiling is used to identify the chromatin status at cis-regulatory regions, promoters and enhancers. The analysis of epigenomic data led to the identification of clusters of enhancers in close genomic proximity, defined as Super Enhancers (SEs), which play an essential role in defining cell identity (24). The identification of SEs is usually performed by looking at the enrichment of different epigenetic features such as lysine 27 acetylation of histone H3 (H3K27ac), the binding of p300 and the binding of master regulator TFs (25, 26). The combinatorial effect of histone marks defines the histone code by providing a more detailed view of epigenomic status at the genomic regulatory regions, and allows better characterization of active sites of transcriptional regulation (27). Whereas, mRNA expression profiling provides a snapshot of the current state of a cell, the understanding of the epigenetic regulation can give a perspective on how this conformation has been reached and could potentially change (28). Immune system adaptation is driven by molecular circuitry in which cell-type specific regulatory regions represent a central component. These core-enhancers are associated with lineage-specific TF binding and they are downstream target of cytokines pathways. Therefore, these genomic regions represent a key regulatory hub of cell-identity and they may be involved in cell plasticity dynamics (29).

In the present study, we used an integrative approach to reconstruct a regulatory network of Th17- and Treg-specific TFs. The network defined using a set of cell type-specific genomic regulatory regions, allowed us to extract putative ERα-regulated enhancers, which are active in these two CD4+ subtypes. Among the identified TFs, RORC, and FOXP3 emerged as candidate targets of estrogenic signaling in Th17 and Treg cells, respectively. We evaluated 17β-estradiol (E2)-induced epigenetic changes at cell type-specific regulatory regions of RORC and FOXP3 loci in Th17 polarizing Peripheral Blood Mononuclear Cells (PBMC). Thus, we monitored the epigenetic status of these regions in PBMCs and purified Th17 and Treg cells derived from RRMS patients and healthy donors during pregnancy. We found that these genomic regions have MS-associated epigenetic signature in cells from pregnant individuals suggesting that they could constitute key regulatory hubs acting as switchers between Th17 and Treg cells in the pathological condition.

## Materials and Methods

### Study Design

This study was designed to investigate the epigenetic profile of Th17 and Treg cells in MS patients during pregnancy. To identify Th17 and T regulatory CSR regulated by ERα, an integrative data analysis was performed on public data sets: first, SEs prediction was combined with chromatin states analysis, and then, a core regulatory network in Th17 and Treg cells based on CSRs and putative ERα binding was reconstructed. Specifically, we focused on *RORC* and *FOXP3* CSRs.

Therefore, peripheral blood of RRMS patients during the third trimester of pregnancy (T3) and in the postpartum period (pp) were collected and analyzed. The institutional review board of each participating center approved the study design and all subjects gave written informed consent. PBMCs from HD were activated under Th17 polarizing condition to test the effects of E2 treatment at pregnancy concentration on the selected CSRs, the mRNA levels of *RORC* and *FOXP3* and the percentage of Th17 and Treg cells. PBMCs from pregnant RRMS patients and HD were analyzed by FACS for Th17 and Treg cells and by Chromatin Immuno Precipitation (ChIP) followed by quantitative PCR (qPCR) for CSRs. The numbers of independent experiments or individuals are given in each figure legend.

### Super Enhancers Prediction

SEs were identified using Rank Ordering of Super Enhancers (ROSE) algorithm (26) in default settings. CD4+CD25–CD45RA+ cells (Naive T), CD4+CD25– T cells (Th), CD4+CD25–IL17+ T cells (Th17), and CD4+CD25+CD45RA+ T cells (Treg) SEs have been defined applying ROSE algorithm on H3K27ac ChIP followed by sequencing (-Seq) datasets of Naive (GSM773004), Th (GSM997239), Th17 (GSM772987), and Treg cells (GSM1056941). Significant H3K27ac ChIP-Seq peaks were defined using MACS2 algorithm version 2.1.0 (30) applied in default settings. Input ChIP-Seq datasets were used as background models for SE and enhancer calling. The list of significant ChIP-Seq peaks was used as input for ROSE algorithm.

### SNPs Analysis

SNPs associated with 41 different diseases were retrieved from GWAS database v2 (31). SNPs were overlapped with SEs from earlier analysis. Enrichment scores were computed generating 1,000,000 random regions of the same length and calculated as:

$$\begin{array}{l} p-value=\frac{1+n^{{}^{\circ}}\;of\;times\;Npermi\;\geq Nobs}{1+n^{{}^{\circ}}\;of\;permutations} \end{array}$$

with:

Nobs = Number of trait-associated SNPs observed to fall in our dataset

Npermi = Number of trait-associated SNPs observed to fall in a randomly generated dataset (*n* = 1,000,000).

### Chromatin States Analysis

Genome segmentation data from Roadmap Epigenomics Project (32) were retrieved from the project website (http://egg2.wustl.edu/roadmap/web_portal) considering the 25-chromatin states model defined on imputed epigenomic data from 127 different cell types. The model is based on imputed data for 12 epigenetic marks (H3K4me1, H3K4me2, H3K4me3, H3K9ac, H3K27ac, H4K20me1, H3K79me2, H3K36me3, H3K9me3, H3K27me3, H2A.Z, and DNase accessibility) predicted by ChromHMM (27). These data report the genomic segmentation computed on each cell type. The segmentation consists in consecutive non-overlapping 200 bp genomic regions annotated with the predicted chromatin state. Segmentation data related to “E039—Primary CD25– CDRA45+ Naive T cells,” “E043—Primary CD25– Th cells,” “E042—Primary IL17+ PMA-I stimulated Th cells,” “E044—Primary CD25+ regulatory T cells” were extracted. The identification of regulatory regions was performed by considering the chromatin states associated with an emission parameter of H3K27ac and H3K4me1 ≥75. Using this threshold, six chromatin states (2_PromU, 9_TxReg, 10_TxEnh5′, 13_EnhA1, 14_EnhA2, 15_EnhAF) were defined as active regulatory states. The segments classified in these states were extracted from the CD4+ segmentation data using an in-house Python script. Then, consecutive genomic segments classified as regulatory were merged defining the regulatory regions set for each CD4+ subtype. To distinguish regulatory regions according to their level of activity among CD4+ subtypes, the chromatin state predicted in each 200 bp fragment composing regulatory regions was compared among CD4+ cell subtypes. If more than half of the fragments within a merged region were classified as active regulatory regions in a specific CD4+ subtype only, the entire region was classified as ARRs in that specific CD4+ subtype. SE-ARRs were obtained overlapping ARRs and SEs using the *intersect* function of Bedtools suite (33).

### Histone Marks Enrichment Analysis

The evaluation of histone marks enrichment within ARRs, SE-ARRs, and CSRs has been performed overlapping selected regions with ChIP-Seq dataset retrieved from Roadmap project using the *intersect* function of Bedtools suite (33). The list of datasets used for this analysis is in Table S1G. Histone marks enrichment in ARRs and SE-ARRs was computed as the mean of replicates over the mean of input datasets in each cell subtype. Histone marks enrichment in CSRs associated genes was computed as the mean of the enrichment in each CSR associated to a single gene.

### Gene Ontology Analysis

Functional and ontological enrichment analysis of genes mapped in proximity of SEs and SE-ARRs was performed using the Genomic Regions Enrichment of Annotations Tool (GREAT) in default mode (34).

### RNA-Seq Analysis

Twenty-five PolyA+ RNA-Seq experiments performed on five CD4+ subtypes isolated from healthy donors were re-analyzed (ArrayExpress Archive of Functional Genomics Data experiment accession: E-MTAB-2319) (35). In detail, sequencing reads of the five replicates of CD4+ Naïve cells (CD4+CCR7+CD45RA+CD45RO–), CD4+ Th1 cells (CD4+CXCR3+), CD4+ Th2 cells (CD4+CRTH2+CXCR3–), CD4+ Th17 (CD4+CCR6+CD161+CXCR3–), and CD4+ Treg cells (CD4+CD127–CD25+) were retrieved and considered for this analysis. Reads were mapped using TopHat v2 (36). The hg19 human genome assembly was used as a reference genome while Gencode v19 as a reference set of gene annotations. Read count was performed using FeatureCounts algorithm and read count tables were normalized with DESeq2 package (37, 38). Normalized read counts were converted to fragments per kilobase of exons per million fragments mapped (FPKM) considering the length of the longest isoform of each gene and the millions of reads. Genes with FPKM > 1 in all five biological replicates available for a CD4+ subtype were considered expressed in that specific subtype. SEs were annotated to CD4+ expressed genes whose TSS was mapped within a distance of 100 Kbp from the center of the nearest SE. Differential expression analysis was performed using DESeq2 package (38). A gene was considered as differentially expressed between two CD4+ subtypes if associated with an adjusted *p* < 0.001. To transform the expression data in Z-score, first, the average expression across the five RNA-Seq replicates of each CD4+ subtype, then the mean expression and the standard deviation across the five CD4+ subtypes were computed.

### Transcription Factor Binding Motif Analysis

A non-redundant list of human Positional Weight Matrices (PWMs) was obtained from the integration of four public PWM databases (HOCOMOCO v10, jolma 2013, CISBP v1.02, Jaspar vertebrates 2016). PWM were selected based on species and quality attributes. Firstly, only human- or mouse-derived PWMs were selected favoring human-TF related matrices. Then, PWMs derived from experimental evidence were preferred to computational inferred ones in case of PWMs concerning the same TF.

TF motifs discovery at ARRs was performed using Find Individual Motif Occurrences software (FIMO) included in the MEME suite for Motif-based sequence analysis (39). A significance threshold of 0.001 on the *p*-value score has been applied for the motif finding analysis.

### Network Reconstruction

Regulatory networks of Th17 and Treg cells were designed considering subtype specific regulatory interactions. Specifically, for each CD4+ subtype, network nodes represent expressed SE-ARR associated genes. A gene was classified as TF using a list of experimentally validated TFs from the Animal Transcription Factor Database (40). Network edges represent regulatory interactions predicted by motif finding analysis performed on SE-ARR sequences using Find Individual Motif Occurrences software (FIMO) included in the MEME suite (39). Then, node inward links connect that node/target with its TF regulators whose binding is predicted at node/target SE-ARRs. Conversely, outward links represent regulatory interaction of a node/TF with its targets by SE-ARRs binding. We called CSRs the subset of SE-ARRs associated with highly differentially expressed TFs between Th17/Treg cells and Naive T cells (DESeq2 FDR adjusted *p* < 1.0 × 10^−7^). Thus, we filtered networks for CSRs, obtaining core regulatory subnetworks. Pairwise gene expression correlation analysis was performed using the 25 FPKM values from CD4+ RNA-Seq analysis (E-MTAB-2319) (35). Pearson linear correlation on each pair of genes was computed. An absolute Pearson coefficient >0.3961 was considered statistically significant for positive or negative correlations (two-tailed *t*-test, *p* < 0.05). Positive and negative correlations were used to represent activatory and inhibitory network links, respectively. For network visualization, Cytoscape version 3.4.0 was used (41). Network analyzer (42) was applied to compute network statistics.

### Patients

Fifteen pregnant MS patients with clinically defined RRMS (mean age 36 ± 4), referred to the academic neurological unit, Department of Clinical and Biological Sciences, University of Turin (IT); AOU Federico II, Regional Multiple Sclerosis Centre, Naples (IT); and Multiple Sclerosis Center, ASST Ospedali Civili di Brescia, Brescia (IT) were enrolled in the study. Inability to express the informed consent, treatment with any RRMS drugs (interferon beta 1a or 1b, glatiramer acetate, tecfidera, teriflunomide, fingolimod, mitoxantron, alemtuzumab), alcohol abuse, cardiopathies, major depression and the concomitance with other autoimmune diseases were exclusion criteria. Fifteen sex and age matched healthy donors, referred to City of Health and Science Academic Hospital, Birth Center Sant'Anna, Turin (IT), were enrolled as the control group. Demographical and clinical characteristics of patients and HDs are outlined in Table 1. Blood samples were collected during routine checkup and processed within 24 h of collection. The institutional review board of the participating centers approved the study design and all subjects gave written informed consent.

**Table 1:** Patients' characteristics.

**Clinical data**	**HD**	**RRMS**
Mean age (years)^a^	32.3 ± 7.4	34.9 ± 4.7
Median EDSS before pregnancy	–	2 (1–5.5)
Median number of relapses before pregnancy		2 (0–2.6)
Median number of relapses after delivery	–	0 (2–0)
Mean number of therapies before pregnancy	–	1.4 ± 1.09

a *Age at T3*.

### PBMCs, Treg, and Th17 Cells Isolation

PBMCs were isolated from whole blood samples by a Ficoll-Paque TM PLUS (GE Healthcare, Milan, IT) density-gradient centrifugation. Treg cells were separated from PBMCs using the CD4+CD25+CD127dim/– Regulatory T Cell Isolation Kit II human (Miltenyi Biotec, GmbH, Germany). This separation consisted in two steps. First, the isolation of CD4+ CD25+ CD127dim/– regulatory T cells was performed with a cocktail of biotinylated antibodies and Anti-Biotin MicroBeads for the depletion of non-CD4+ and CD127high cells by separation over a MACS® Column, which is placed in the magnetic field of a MACS® Separator (Miltenyi Biotec, GmbH, Germany). In the second step, the flow-through fraction of pre-enriched CD4+CD127dim/– T cells was labeled with CD25 MicroBeads for subsequent positive selection of CD4+CD25+CD127dim/– regulatory T cells. Negatively selected fraction of CD4+CD25– T cells was collected for the next separation of Th17 cells. This cell fraction was stimulated with 50 ng/ml Phorbol-12-myristate-13-acetate (PMA) and 500 ng/ml Ionomycin (Sigma Aldrich) at 37°C for 4 h to induce cytokines production. Th17 cells were then separated using IL-17 Secretion Assay-Cell Enrichment and Detection Kit human (Miltenyi Biotec, GmbH, Germany). PMA-ionomycin stimulated cells were mixed with the provided IL-17 Catch Reagent and incubated for 45 min at 37°C to allow the reagent to bind the positive, secreting cells. IL-17–secreting cells were subsequently labeled with a second PE-conjugated IL-17–specific antibody and finally magnetically labeled with Anti-PE MicroBeads UltraPure and separated over a MACS® Column.

### *In vitro* Th17 Cells Polarization

Isolated PBMCs from female healthy donors (HD) (18–45 years old) were cultured in RPMI 1640 medium containing 10% estrogen deprived Fetal Bovine Serum (FBS), 2% HEPES, 1% Glutamax, and 1% Gentamicin. They were activated with plate-coated anti-CD3 (10 μg/ml) and soluble anti-CD28 monoclonal antibodies (mAbs) (1μg/ml; BD Biosciences, San Diego, CA) for 3 days in the presence of IL-23 (50 ng/ml; R&D Systems) plus anti–IFNγ (100 ng/μl; Biolegend, San Diego, CA) as previously described (*3*). At day 0, cells were treated with 17β-estradiol (E2) 35 ng/mL or vehicle (ethanol) in concomitance with Th17 polarizing cytokines.

### Flow Cytometry Analysis

PBMCs were stained for Treg cells with anti-CD4, anti-CD25, and anti-CD127 mAbs (Biolegend, San Diego, CA) on the cell surface. For detection of the transcriptional factor FoxP3, cells were fixed with Fixation and Permeabilization Buffers (eBioscience, San Diego, CA) and were then stained with anti-FoxP3 mAb (eBioscience, San Diego, CA). The expression of IL-17 was analyzed by intracellular cytokine staining. PBMCs were cultured in Iscove's Modified Dulbecco's Medium (BioWhittaker, Walkersville, MD) supplemented with 10% Fetal Bovine Serum (FBS, Invitrogen, Carlsbad, CA) and stimulated for 5 h with Phorbol 12-myristate 13-acetate PMA (50 ng/ml) and ionomycin (500 ng/ml) in the presence of Brefeldin A (BFA, 10 μg/ml, Sigma-Aldrich, St. Louis, MO). Cells were first stained for the surface antigen CD4, (Biolegend, San Diego, CA) and then fixed with 4% paraformaldehyde, permeabilized with 0.5% saponin, followed by intracellular staining with anti-IL-17 mAb (Biolegend). ERα expression on Th17 and Treg cells was detected by staining with ERα mAb (LSBio, Seattle, WA). The ERα specific cell-associated mean fluorescence (ΔMFI) was calculated by subtracting the MFI of cells stained with control isotype IgG from that of cells stained with ERa mAb. Stained PBMCs were acquired on a BD Accuri^TM^ C6 Cytometer (BD Biosciences, San Jose, CA) and analyzed with FlowJo software (Ashland, OR).

### Chromatin Immunoprecipitation Assay

We adapted a ChIP protocol optimized for a small amount of chromatin (43). PBMCs and purified Treg and Th17 cells were incubated with 1% formaldehyde in PBS 1X for 10 min at 37°C. The crosslinking reaction was stopped by adding glycine at a final concentration of 125 mM followed by incubation at room temperature (RT) for 5 min. PBMCs nuclear extracts were then obtained with a two-step lysis procedure using Cell Lysis Buffer (5 mM Pipes pH 8.0, KCl 85 mM, NP40 0.5%) and Nuclei Lysis Buffer (SDS 1%, EDTA 10 mM, Tris-HCl pH 8.1 50 mM) both added with 1X protease inhibitor cocktail (Sigma-Aldrich) and 1 mM phenylmethylsulfonylfluoride (PMSF, Sigma-Aldrich). In the case of purified Th17 or Treg cells, only Nuclei Lysis Buffer step was performed. Cell lysates were incubated on ice for 10 min and then sonicated in two different ways according to the starting sample. Chromatin from PBMCs was fragmented by 20 sonication cycles consisting of 20″ on and 50″ off using Sonopuls HD2070 sonicator (Bandelin). Th17 and Treg chromatin were fragmented for 30 pulses 30″ON/30″OFF high with Bioruptor Twin (Diagenode). A small fraction of chromatin was decrosslinked with 50 μg of Proteinase K (Thermo Fisher Scientific) and DNA was purified with phenol chloroform (Ambion, Applied Biosystems), followed by ethanol precipitation. Chromatin fragmentation was checked by electrophoretic separation of DNA on a 1.2% agarose gel. One microgram of sonicated chromatin was diluted in IP buffer to a final volume of 120 μl for each immunoprecipitation, and incubated with 0.5 μg of antibodies against human H3K4me3 (Diagenode), H3K27me3 (Active Motif), H3K4me1 (Diagenode), H3K27ac (Active Motif), ERα (HC-20) X, and ERα (H-184) X (Santa Cruz Biotechnology) in a BSA precoated Corning™ Falcon™ Polystyrene 96-well microplate (Thermo Fisher Scientific). Lysates with Ab were incubated at 4°C overnight on an orbital shaker. Samples with IgG antibody (Abcam) were run in parallel as negative controls. The following day, 30 μl of 50% Protein A Sepharose^TM^ 4 Fast Flow (GEHealthcare) slurry was added and incubated for 2 h at 4°C to purify the immune complexes. Proteins and DNA complexes non-specifically associated with beads were removed by sequential washes with low-salt buffer (0.1% SDS, 1% Triton X-100, 2 mM EDTA, 20 mM Tris-HCl pH8.0, and 150 mMNaCl), high-salt buffer (0.1% SDS, 1% Triton X-100, 2 mM EDTA, 20 mM Tris-HCl, pH 8.0, and 500 mMNaCl), LiCl washing buffer (0.25 M LiCl; 1% deoxycholate sodium salt, 1 mM EDTA, 10 mM Tris-HCl pH8.0, and 1% NP-40) and twice with Tris-EDTA buffer (10 mM Tris-HCl pH 8.0, 1 mM EDTA). Samples were maintained at 4°C for 5 min on an orbital shaker each wash. The immunoprecipitated DNA-protein complexes were purified by 10% Chelex® 100 Resin (BIO-RAD) for 10 min at 95°C. Proteins were digested incubating each sample with 20 μg of Proteinase K (Thermo Fisher Scientific) for 30 min at 55°C and then 10 min at 95°C to obtain Proteinase K inactivation and DNA purification. The resulting purified DNA was used for following qPCR analysis.

### Total RNA Extraction

Total RNA was extracted using TRIzol® Reagent (Invitrogen) according to the manufacturer protocol. Concentration of RNA in samples was measured by NanoDrop 1,000 Spectrophotometer (Thermo Fisher Scientific). Extracted RNA were then treated with ezDNase™ Enzyme (Thermo Fisher Scientific). DNA-free RNA was reverse-transcribed into complementary DNA (cDNA) with SuperScript™ IV VILO™ Master Mix (Thermo Fisher Scientific). Resulting cDNA was used for qPCR analyses.

### Real Time Quantitative PCR

Real Time PCR was performed using 7300 Real Time PCR System (Applied Biosystems) and the iTaq Universal SYBR Green Supermix (Biorad) in 96-wells multiwell plates (Applied Biosystems). FOXP3 and RORC mRNA expression was determined using QuantiTect Primer Assays (QIAGEN, Hilden, Germany). Relative quantification of mRNA was normalized on 18 s mRNA level. ChIP signals were normalized on input samples (10% of total chromatin used per IP) and expressed as enrichment of specific binding over the control non-specific IgG binding. Primers for ChIP-qPCR analysis of promoter and enhancers were designed using Primer3Plus software. Designed primer were tested with *in silico* PCR tool (https://genome.ucsc.edu/) in order to check specificity of amplification during PCR reaction and with AnnHyb software (http://bioinformatics.org/annhyb/) to verify self-hybridization and dimer formation of primers. Primers were synthesized by Bio-Fab Research (Rome, Italy) (Table S3).

### Statistical Analysis

Statistical analyses were performed using GraphPad Prism 5 (GraphPad Software, San Diego, CA). Student's *t-*test for paired values and one-way analysis of variance (ANOVA) followed by Bonferroni multiple comparison post-test, were used. *P* < 0.05 was considered to be significant.

## Results

### Definition of Active Regulatory Regions Within Super Enhancers of CD4+ T Cell Subtypes

**Figure 1:**
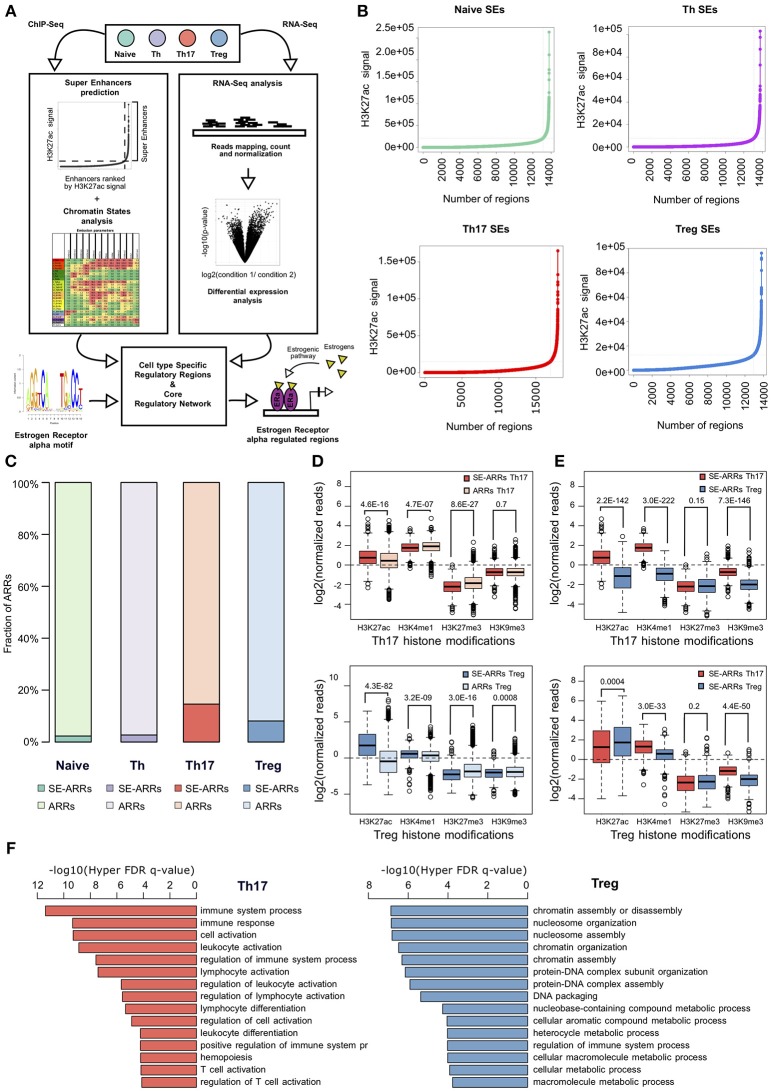
Active regulatory regions within super enhancers of CD4+ T cell subtypes **(A)** Workflow representation of our approach for data integration. SEs prediction in CD4+ T cell subtypes and chromatin states analysis were used for identification of active regulatory regions (left side). Overlap between these regions defines SE-ARRs. DE Gene expression analysis led to the identification of main TFs involved in Th17 and Treg lineage determination (right side). Finally, we reconstructed a SE-ARRs-associated TFs regulatory network in Th17 and Treg cells. By this analysis, we identified putative targets of ERα-mediated regulation in Th17 and Treg cells. **(B)** Prediction of SEs in Th17, Treg, Naive T, and Th cells by Rank Ordering of Super Enhancers (ROSE) algorithm. Line plot reports the cumulative number of enhancers identified in Th17 and Treg cells as function of the number of H3K27ac ChIP-Seq reads over the input dataset. Vertical lines represent the threshold over which H3K27ac signal intensity defines SEs. **(C)** Bar plot shows the fraction of ARRs overlapping SEs in Th17 and Treg. **(D,E)** Box plot shows the log2 normalized H3K27ac, H3K4me1, H3K27me3, and H3K9me3 ChIP-Seq signal measured in Th17- **(D)** and Treg- **(E)** SE-ARRs. On right panels SE-ARRs comparison with ARRs. *P*-value by Wilcoxon Rank-sum test. **(F)** Bar plot shows top 15 most significant Gene Ontology Biological Processes enriched by Genomic Regions Enrichment of Annotations Tool (GREAT) for genes mapped in proximity of Th17 (left) and Treg (right) SE-ARRs.

Genomic regulatory regions are integrative hubs for cellular pathways activated upon environmental stimuli. Since we were interested in the identification of putative genomic targets of estrogens signaling in Th17 and Treg cells, ERα modulated chromatin regulatory hubs were identified by using an integrative analysis of epigenomic and transcriptomic data. We designed a computational approach composed of four consecutive Next Generation Sequencing (NGS) data integration steps: (i) SEs prediction in CD4+ T cell subtypes, (ii) chromatin states analysis for identification of active regulatory regions, (iii) overlap between these regions and SEs detected in Th17 and Treg cells, (iv) reconstruction of a core TFs regulatory network of Th17 and Treg cells and identification of putative ERα targets (Figure 1A).

We predicted SEs using public H3K27ac ChIP-Seq data of human CD4+CD25–CD45RA+ cells (Naive T), CD4+CD25– T cells (Th), CD4+CD25–IL17+ T cells (Th17), and CD4+CD25+CD45RA+ T cells (Treg) from the Roadmap Epigenomics Project (30), identifying 658, 676, 999, and 851 SEs in Naive T, Th, Th17, and Treg cells, respectively (Figure 1B and Table S1A). Interestingly, Gene Ontology (GO) analysis of genes mapped in proximity of SEs showed an association with “immune response” and “regulation of immune system” processes (Table S1B). We also evaluated the enrichment of Single Nucleotide Polymorphisms (SNP) associated to a set of 41 diseases, within Th17 and Treg SEs. Autoimmune-disease-associated SNPs, overlapped more often with Th17 and Treg SEs than with a random set of regions of the same length. This enrichment is stronger for autoimmune-disease-associated SNPs in respect to the control group of other-disease-associated SNPs (Figure S1A).

To identify Active Regulatory Regions (ARRs) in SEs of Th17 and Treg cells, we analyzed chromatin states data predicted by ChromHMM (44) in the aforementioned CD4+ T cell subtypes. This model consists of 25-chromatin states model based on imputed data for 12 epigenetic marks defined for 127 cell types and provides a 200 bp human genome segmentation with the corresponding predicted functional annotation. Using this data, we selected a subset of 65,581 genomic regions characterized by an enrichment of H3K27ac and lysine 4 mono-methylation of histone H3 (H3K4me1) whose co-occurrence defines active enhancers (45). To distinguish these regions according to their level of regulatory activity among CD4+ T cells, we compared their epigenetic state (see Methods for details) and found 4,610 (7.03%), 7,508 (11.45%), 4,720 (7.20%), and 5,608 (8.55%) ARRs exclusive to naive T, Th, Th17, and Treg cells, respectively (Figure S1B and Table S1C). Then, to further isolate ARRs characterized by the highest predicted regulatory activity, we overlapped ARRs with predicted SEs in these cell subtypes. The 2.27, 2.73, 14.60, and 8.10% of naive-, Th-, Th17-, and Treg-ARRs, respectively, overlapped with SE regions (Figure 1C and Table S1D). As expected, SE-overlapped ARRs (SE-ARRs) showed significantly higher levels of H3K27ac compared with ARRs (Figure 1D). Moreover, the comparison of Th17 and Treg SE-ARRs underlines that H3K27ac in SE-ARRs has a cell-type specific enrichment (Figure 1E). Gene Ontology (GO) analysis for genes mapped in proximity of Th17 SE-ARRs showed an association with immune system and inflammatory processes, whereas Treg SE-ARRs are associated with chromatin remodeling and metabolism (Figure 1F and Tables S1E,F).

### Reconstruction of Cell Type-Specific Regulatory Networks Identifies ERα-Regulated Genomic Regulatory Regions in Th17 and Treg Cells

**Figure 2:**
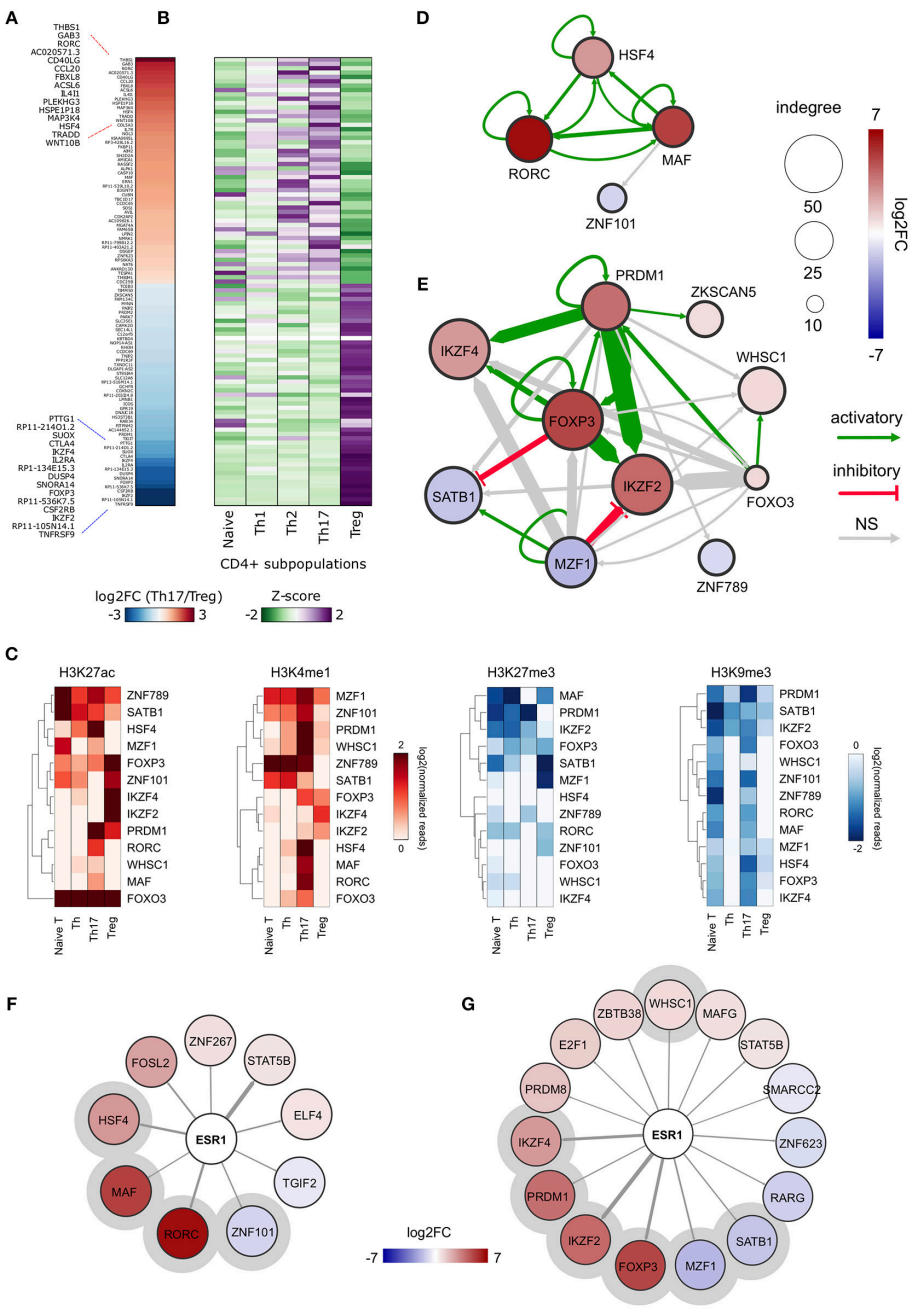
ERα-regulated genomic regulatory regions inTh17 and Treg cells. **(A)** Heat map representing the log2FC of expression computed between Th17 and Treg RNA-Seq data. Only data of SE-ARRs associated significantly DE genes between the two CD4+ cell type are reported. Genes are sorted by decreasing Th17/Treg log2FC. **(B)** Heat map representing the gene expression specificity computed in each CD4+ population as Z-score of expression. Purple colors represent specifically overexpressed genes while green color specifically underexpressed genes. **(C)** Heatmap shows log2 normalized H3K27ac, H3K4me1, H3K27me3 and H3K9me3 ChIP-Seq signal measured in CSRs associated nodes from Th17 and Treg core regulatory networks. Hierarchical clustering shows differences between the epigenetic asset of Treg- and Th17- CSRs. **(D,E)** Th17 **(D)** and Treg **(E)** core regulatory networks. Core regulatory networks are reconstructed by filtering total regulatory networks for SE-ARRs associated TFs with a significant fold change (DESeq adjusted *p*-value < 1 × 10^−7^). Node size is scaled to indegree values. Node color represents log2 fold change expression of Th17/Naive CD4+ cells and Treg/Naive CD4+ cells, respectively. Edge thickness is scaled to the sum of predicted TF binding sites at target-associated CSRs. Edge color represents positive (green) or negative (red) regulation inferred by Pearson correlation analysis between regulator and target gene expression. Positive and negative correlations are used to represent activatory and inhibitory network edges, respectively. Since PWMs are not available for all TFs, some interactions could not be predicted. **(F,G)** Networks show predicted ERα binding at SE-ARRs associated TFs in Th17 **(F)** and Treg **(G)** cells. Edge thickness is proportional to the number of ERE identified at target SE-ARRs. Node color represents log2 fold change expression of Th17/Naive CD4+ cells and Treg/Naive CD4+ cells, respectively. Node size is fixed. ERα targets included also in respective core regulatory network are highlighted with a gray circle.

In order to obtain an overview of gene expression profiles associated to SE-ARRs in Th17 and Treg cells, we re-analyzed raw data from a paired-end tag poly (A+) RNA-Seq datasets performed on purified CD4+ T cells, including Th17 and Treg cells, from five human healthy donors (35). We found 1,291 significantly Differentially Expressed (DE) genes between Th17 and Treg cells, 147 of which associated to SE-ARRs mapped within a distance of 100 kbp (Figure 2A and Table S2A). Comparison of the expression specificity among CD4+ T cells highlighted that upregulated genes in Treg cells were more specific of this CD4+ subtype, while upregulated genes in Th17 cells were similarly expressed in Th1 and Th2 subtypes (Figure 2B). Interestingly, among these genes, the highest DE TF-coding genes associated with SE-ARRs were *RORC, HSF4*, and *MAF* in Th17 cells, and *IKZF2, FOXP3*, and *IKZF4* in Treg cells (Table S2A).

To identify putative regulatory interactions between SE-ARRs associated TFs, we explored the sequence of SE-ARRs for the binding motif of a list of human TFs (see Methods for details). Results of this analysis were used to reconstruct a core TF regulatory network in which the indegree of nodes, representing TF-coding genes, is given by the number of significant TF binding motifs enriched at gene-associated SE-ARRs. Conversely, the outdegree of nodes is the sum of predicted TF bindings to other gene-associated SE-ARRs (46) (Figure S1C). We extracted information on key candidate TFs involved in Th17 or Treg lineage determination by computing the differential gene expression between Th17/Naive and Treg/Naive CD4+cells. We identified 4 and 10 SE-ARR-associated DE TFs (FDR adjusted *P* < 1.0 × 10^−7^) in Th17/Naive and Treg/Naive comparison respectively (Tables S2B–E). We used these TFs to create subnetworks of the total regulatory networks (Figures 2D,E). Then, we enriched these subnetworks with activation and inhibition regulators inferred by a correlation analysis of gene expression (Figures 2D,E and Table S2F). Our network reconstruction highlighted *RORC, MAF*, and *HSF4* as nodes with highest indegree in the Th17 network, and *FOXP3, IKZF2, IKZF4, PRDM1*, and *SATB1* as core regulated genes in Treg cells (Figures 2D,E).

Interestingly, the subset of SE-ARRs associated with these DE TFs show a cell-type specific enrichment of epigenetic marks associated with active enhancers. Hierarchical clustering analysis of single histone modification within these SE-ARRs discriminates the different CD4+ T cells subtypes (Figure 2C). Hence, we called these regions Cell-type Specific Regulatory regions (CSR).

Finally, since our main interest was to identify targets for genomic pathway of estrogens, we sought for the enrichment of estrogen response elements (ERE) within Th17 and Treg SE-ARRs. We found an enrichment of ERE in SE-ARRs associated to 46 TFs identified in Th17, and to 65 TFs in Treg cells. Among these TFs, 9 and 15 are DE (FDR adjusted *P* < 1.0 × 10^−3^) in Th17/Naive and Treg/Naive cells, respectively (Figures 2F,G and Tables S2G,H).

Collectively, this analysis shows CSR-associated TFs in Th17 or Treg cell differentiation. Moreover, it revealed *RORC* and *FOXP3* as first major candidates of ERα-mediated regulation.

### E2 Impairs Th17 *in vitro* Polarization Inducing Chromatin Remodeling at FOXP3- and RORC-CSRs

**Figure 3:**
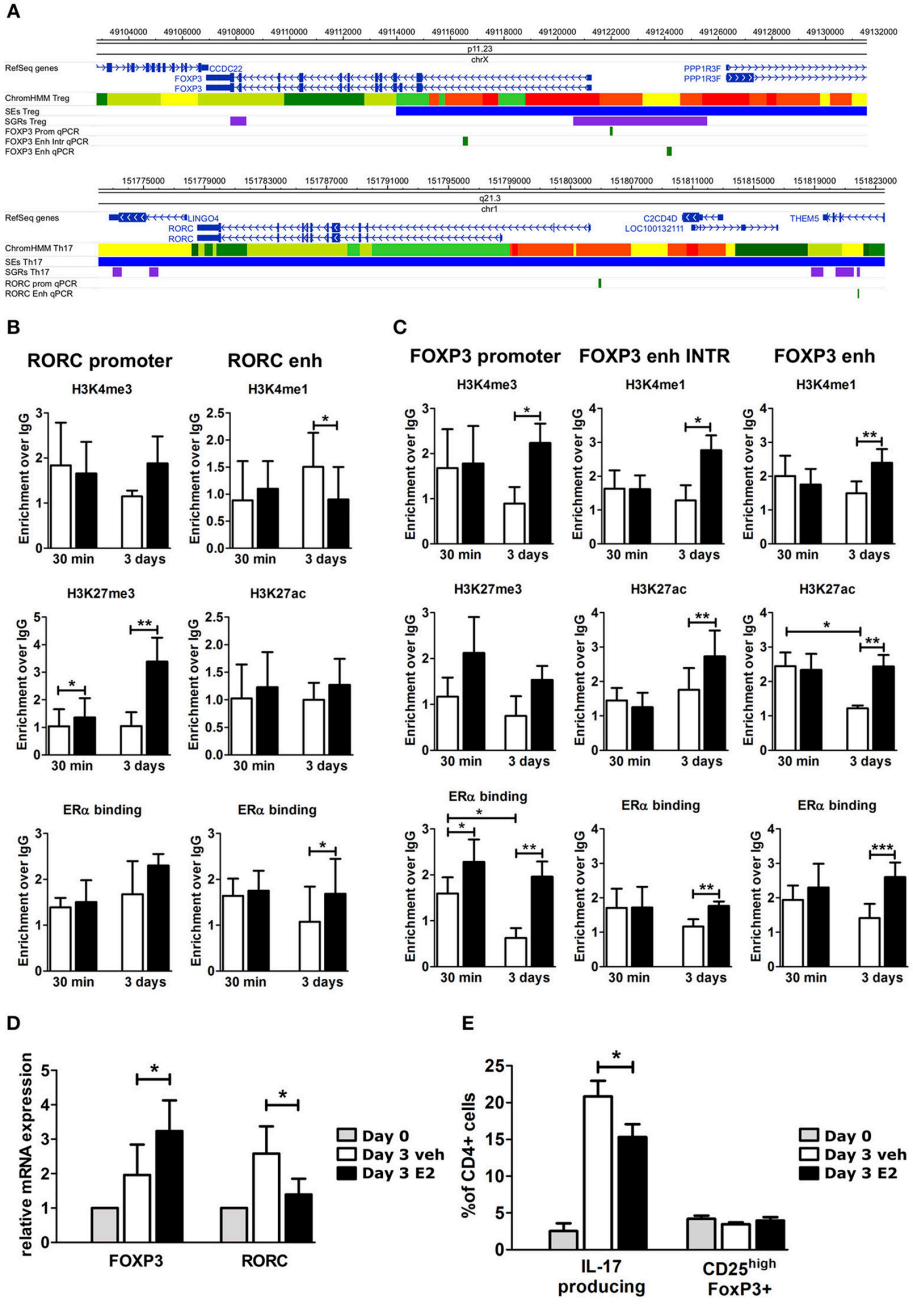
E2 impairs Th17 polarization inducing chromatin remodeling at CSRs. **(A)** UCSC Browser of human FOXP3 locus and RORC locus. First colored bars represent the chromatin states (e.g., yellow segments are classified as active enhancers). Blue bars are predicted SEs, purple bars are ARRs and green bars are the regions that we analyzed. **(B–E)** PBMCs from five HD were polarized under Th17 polarizing conditions with or without E2 treatment. ChIP-qPCR analysis of H3K4me3, H3K27me3, H3K4me1, H3K27ac, and ERα at RORC- **(B)** and FOXP3- **(C)** CSRs. Columns represent the enrichment of the immunoprecipitation over non-specific IgG and normalized for input chromatin at 30 min and 3 days of stimulation. FOXP3 and RORC mRNA expression **(D)**, and FACS analysis of Th17 and Treg cells in CD4_lymphocytes **(E)** stimulated for 3 days. *p < 0.05, **p < 0.01, and ***p < 0.001 represent the statistical significance.

To understand the effects of E2 on Th17 cells during pregnancy, we activated peripheral blood mononuclear cells (PBMC) from female healthy donors (HD) *in vitro* under Th17 polarizing conditions in the presence and absence of E2 at pregnancy concentration (35 ng/ml). Figure 3A shows *FOXP3* and *RORC* loci, with associated CSRs derived from previously described bioinformatic analysis. We designed primers within these regions, and in two other biologically relevant regions: *FOXP3* intronic Conserved non-coding sequence 2 (CNS2) (47) and RORC promoter.

Chromatin immunoprecipitation assay followed by qPCR (ChIP-qPCR) was performed against typical histone marks of promoters and enhancers and ERα binding. At *RORC* locus, E2 treatment increases H3K27me3 enrichment at gene promoter and ERα binding at the enhancers, whilst decreasing H3K4me1 levels at the enhancer (Figure 3B). By contrast, at *FOXP3* locus, E2 treatment increases H3K4me3 enrichment at gene promoter, H3K4me1 and H3K27ac enrichment at the enhancers and the binding of ERα in all of the tested regions (Figure 3C). The epigenetic changes induced by E2 treatment at *FOXP3* and *RORC* loci reflect an enhanced *FOXP3* and an impaired *RORC* mRNA expression (Figure 3D). Consistently, E2 treatment induces a significant inhibition of Th17 cells expansion and a slight increase of Treg cells that return to similar levels to those before polarization (Figure 3E).

Altogether, these data indicate that E2 treatment impairs Th17 expansion and induces a chromatin remodeling at CSRs involved in Th17 and Treg subtype definition.

### Pregnancy-Associated Epigenetic Signature at CSRs in Th17 and Treg Cells of MS Patients

**Figure 4:**
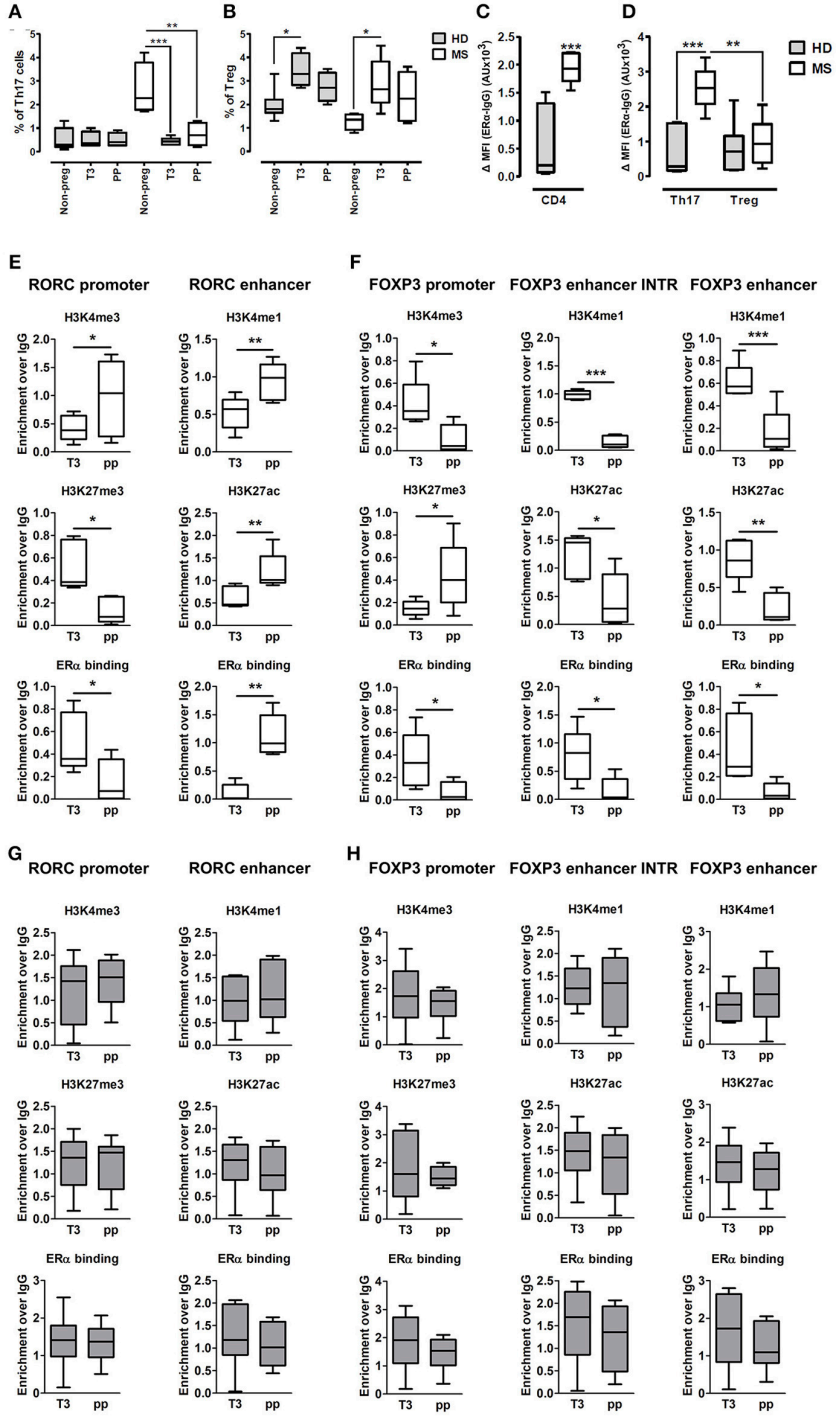
Epigenetic changes at FOXP3 and RORC loci in PBMCs from MS patients during pregnancy. **(A,B)** Th17 and Treg cells percentage, evaluated by FACS, in the PBMCs of HD (gray bars) and MS patients (white bar) non-pregnant, during the T3 and in the pp. **(C,D)** Expression of ERα, evaluated by FACS, on total CD4+ T cells, Th17, and Treg cells from HD and MS patients. Graph shows ERα specific cell-associated mean fluorescence (ΔMFI). **(E–H)** ChIP-qPCR analysis of H3K4me3, H3K27me3, H3K4me1, H3K27ac, and ERα binding on PBMCs derived from MS patients **(E,F)** and HD **(G,H)** during T3 and in the pp. Boxes, with mean, minimum and maximum, represent the enrichment of the immunoprecipitation over non-specific IgG and normalized for input chromatin. *p < 0.05, **p < 0.01 and ***p < 0.001 represent the statistical significance.

Fifteen pregnant RRMS patients and fifteen pregnant healthy donors (HD) were studied during the third trimester of pregnancy (T3) and the postpartum period (pp). The epidemiological and clinical characteristics of these subjects are summarized in Table 1. In the peripheral blood, we observed a significant reduction of Th17 cells in the T3 (0.45% ± 0.06) and in the pp (0.73% ± 0.19) compared with active non-pregnant RRMS (2.6% ± 0.56), whereas no difference was detected in HD (Figure 4A). Treg cells increase significantly in the T3 both in HD (3.42% ± 0.23) and in RRMS (2.86% ± 0.43) compared with non-pregnant HD (1.9% ± 0.24) and active RRMS (1.27% ± 0.17, Figure 4B). Interestingly, CD4+ T cells from RRMS patients expressed significantly higher levels of ERα compared with HD (Figure 4C), and this feature was peculiar of Th17 cells but not of Treg cells, as ERα was expressed at the same level both in Treg cells from HD and RRMS (Figure 4D). These data suggest that estrogens may affect circulating CD4+ T cells, especially Th17 cells in RRMS.

To understand if chromatin remodeling occurs in lymphocytes from RRMS patients during pregnancy, *RORC* and *FOXP3* CSRs were tested for histone marks and ERα binding in PBMCs derived from RRMS patients during T3 and pp. At RORC promoter, ERα binding is higher during T3 and correlates with a higher H3K27me3 and a lower H3K4me3 during T3. During pp, we observed an increment of ERα binding at *RORC-*associated enhancer, going on with higher H3K4me1 and H3K27ac enrichment (Figure 4E). ERα binding at *FOXP3* promoter and enhancers is higher in T3 compared with pp. This goes along with H3K4me3 increment at the promoter and H3K4me1 and H3K27ac enrichment at the enhancers of FOXP3 during T3 compared with pp. Simultaneously, the fall of ERα binding during pp is associated with a higher H3K27me3 enrichment at *FOXP3* promoter (Figure 4F). None of these epigenetic changes occurs at *RORC* (Figure 4G) and *FOXP3* (Figure 4H) CSRs in PBMCs derived from HD. These results suggest a MS specific epigenetic profile characterized by activation of *FOXP3* CSRs and inactivation of *RORC* CSRs during T3 and by the activation of *RORC* CSRs and the inactivation of *FOXP3* CSRs during the pp.

**Figure 5:**
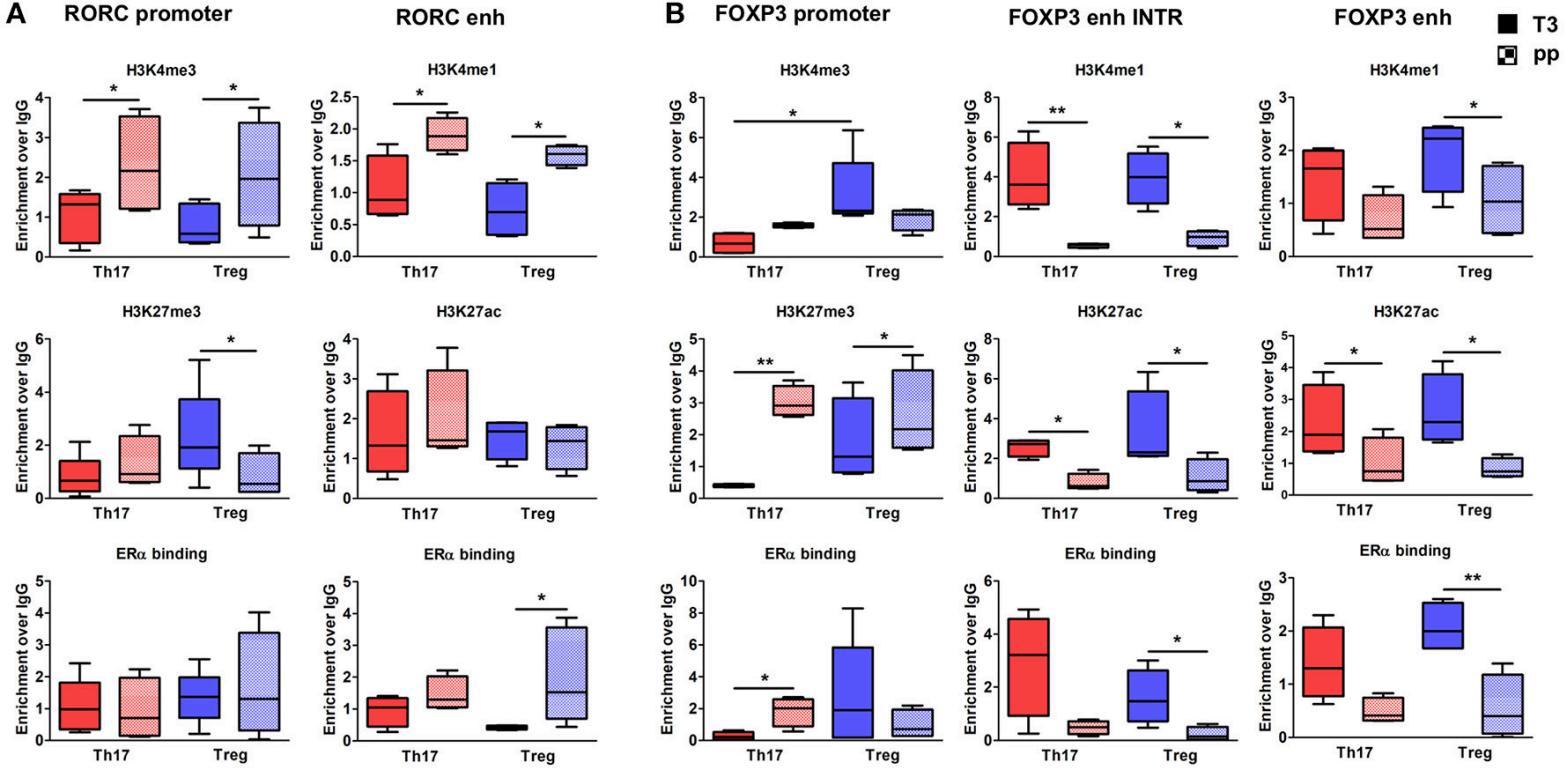
Epigenetic changes at FOXP3 and RORC loci in Treg and Th17 purified from MS patients during pregnancy. ChIP-qPCR analysis of H3K4me3, H3K27me3, H3K4me1, H3K27ac, and ERα binding performed at *RORC*- **(A)** and *FOXP3*-CSRs **(B)** in Th17 (red) and Treg (blu) cells, derived from MS patients during third trimester of pregnancy T3 (filled texture) and during the post-partum pp (squared texture) phase. Boxes, with mean, minimum and maximum, represent the enrichment of the immunoprecipitation over non-specific IgG and normalized for input chromatin. *p < 0.05 and **p < 0.01 represent the statistical significance.

To better clarify in which cell type these epigenetic variations occur, we tested the epigenetic changes in all the selected *FOXP3* and *RORC* CSRs in purified Th17 and Treg cells from 6 RRMS pregnant patients during T3 and pp. In purified Th17 cells, we observed an enrichment of H3K4me3 at *RORC* promoter and H3K4me1 at the enhancer in the pp suggesting activation of *RORC* (Figure 5A). Surprisingly, we observed the same epigenetic variations at *RORC* locus in purified Treg cells (Figure 5A). In this subtype, the enrichment of H3K4me1, H3K27ac, and a higher ERα binding at *FOXP3* enhancers were observed during T3, whereas H3K27me3 level at *FOXP3* promoter increase in the pp, indicating activation of *FOXP3* during T3 and its inactivation during pp (Figure 5B). The activation of *FOXP3* during T3 correlates with higher binding of ERα at FOXP3 enhancers. Once again, the same variations were observed at *FOXP3* locus in Th17 cells (Figure 5B). The observation of the same epigenetic variation in both Th17 and Treg cells suggest a mutual plasticity of these cells that could be regulated mainly by estrogens.

Overall, epigenetic analysis of PBMCs and purified Th17 and Treg cells indicate that *FOXP3 CSRs* were activated while *RORC CSRs* were inactivated during T3 of RRMS patients.

## Discussion

Despite the numerous evidence that estrogen has beneficial effects on the clinical signs of MS and EAE and the emerging results of which are E2-responsive target cells in the EAE (10, 48, 49), little is known about the molecular signaling above E2. In this study, we identified a peculiar epigenetic profile of Th17 and Treg cells of MS during pregnancy that could be associated to ERα activation. ERα expression and signaling in encephalitogenic CD4+ T cells was reported to be required for sustained EAE protection (10). Here, we show that RRMS CD4+ T cells, and in particular Th17 cells, express higher levels of ERα, making them supposedly more responsive to estrogen level variations. This could be considered a peculiar characteristic of pathogenic Th17 cells of MS patients that, as broadly demonstrated, display a typical expression of cytokines, chemokines, transcription factors and membrane receptors that are characteristics of pathogenic Th17 cells but not of Th17 cells involved in the response to pathogens (50). In the EAE model, Th17 cells were shown to be a target for E2 that resulted in the inhibition of encephalitogenic Th17 cells expansion (51); the mechanisms of this anti-inflammatory effects of E2 involved both by a direct action on Th17 cells (10) and the expansion of Treg cells (13, 15), induction of tolerogenic dendritic cells and recently, regulatory B cells (52). Similar to the EAE model and previous MS studies (53, 54), here we show that in the T3 of pregnancy, where estrogens reach the highest levels, Th17 cells strongly decreased, whereas Treg cells increased; such results could be indicative of a less inflammatory environment in MS patients during pregnancy.

From clinical point of view, pregnancy is accepted to be a period in which relapses decrease significantly, especially in the third trimester as explored in several clinical studies (6, 7, 55, 56). From an immunological point of view, Immune system is not the main target for sex hormones, however the high level of estrogens during pregnancy exerts its role on immune system adaptation contributing to immunotolerance, such as hematopoietic and Treg cells proliferation (57, 58). On the contrary, the postpartum phase is characterized by a strong drop in estrogens level, with immunomodulation lost (59). These two phases represent a unique opportunity for comparison, as pregnancy maximizes the immune cell subtypes differences between third trimester and post-partum resembling, respectively, remission and relapse phases of MS disease. Pregnancy immunotolerance in MS, with a dominance of Treg cells over Th17 cells respect to postpartum is associated with the physiological immunotolerance (53, 60).

Although Th17 and Treg cells represent two CD4+ T cell subsets with opposing principal functions, these cell types are functionally connected; for example, TGF-β links the development of Th17 cells to that of Treg cells: TGF-β indeed induces the differentiation of Treg cells but in combination with IL-6 or IL-21 promotes the induction of Th17 cells and inhibit Treg cells differentiation (61). At the molecular level, FOXP3, the master regulator TF of Treg cells, could bind physically to RORC, the master regulator TF of Th17 cells, to antagonize its function (62). Furthermore, other factors, such as retinoic acid (RA), aryl hydrocarbon receptor (AHR) or hypoxia inducible factor 1α (HIF-1α) can regulate the balance between Th17 and Treg cells (63). Plasticity has been observed between both antagonistic cell type: Th17-like Treg cells, i.e., FoxP3 Treg cells also expressing IL-17, has been reported (64, 65) and seems to depend on epigenetic modifications (66). The role of the chromatin landscape is indeed important in the context of TF action and cellular plasticity, as the chromatin state deeply influences TF binding. Here, by using a bioinformatics integrative approach, we selected the putative genomic regulatory regions that may be a target for ERα signaling in the epigenetic control. The observation that the same epigenetic variation occurs in both purified Th17 and Treg cells suggests a mutual plasticity of these cells that could be regulated mainly by estrogens.

ERα cistrome has been extensively studied in breast and endometrium: genome wide data sequencing of ERα binding, integrated with epigenetic marks and chromatin long range interactions data allow for the prediction of ERα action. One of the most important results derived from integrative analysis in breast cancer experimental models is that EREs and ERα binding are enriched at SEs (67). Furthermore, the crosstalk between ERα and inflammatory signaling plays a role in the endocrine resistance of breast carcinoma. ERα phosphorylation and cofactor recruitment by cytokine stimulation induces a constitutive ERα-dependent activation of gene expression and proliferation that is involved in cancer progression and resistance to endocrine therapy (68, 69). ERα, bound to DNA at distal genomic regulatory regions of target genes, interacts with transcription factors and recruits coactivators or corepressors that mediate the association with enzymes able to remodel chromatin (70). Orchestrating chromatin architecture, ERα may mediate epigenetic modifications at chromatin hubs in CD4+ T cells, influencing their differentiation and plasticity, as well as it does in its main target tissues. On this way, ERα may act as cooperative transcription factor in T cell epigenome dynamics for the environment adaptation (71).

Next Generation Sequencing data allow the capture of different -omics information, and multilevel studies integration can provide an upgrade of knowledge about immune system cells. Integrative data analysis confers novel functions to specific genomic regions that are hubs of gene regulatory circuitry by recruiting transcriptional complexes. Molecular mechanisms underlying transcriptional regulation guided our integrative analysis of epigenomic and transcriptomic data. On these bases, we reconstructed a regulatory network in human Th17 and Treg cells, highlighting CSR-associated TFs that cooperate for cell identity determination. Network reconstruction has already been explored in Th17 mouse cells combining -omics data integration with KO or innovative perturbation tools (23, 72). Recently, even single-cell RNA-Seq has been used to investigate molecular mechanisms governing heterogeneity and pathogenicity of Th17 cells (73). Regarding Treg cells, network analysis approach has, to date, never been explored.

Concerning Th17 cells, our core regulatory network shows similarities with previously mentioned mouse networks. The three upregulated TFs that stand out from our network are *RORC, MAF*, and *HSF4. RORC* is the master regulator of the Th17 lineage. It has yet been shown that E2 recruits a repressor on *RORC* promoter EREs via ERα, thus inhibiting *RORC* expression and Th17 differentiation (48). The role of *MAF* in Th cells and autoimmunity has been extensively explored. Gustafsson et al. proposed MAF, together with *GATA3* and *MYB* as early regulators of T cell–associated diseases (74). These TFs are enriched in autoimmunity-associated polymorphisms and DE between Th1 and Th2 subtypes at early stages of differentiation. In addition, they show DE of splice variants during asymptomatic and symptomatic stages of seasonal allergic rhinitis. A MAF-associated long intergenic non-coding RNA *(linc)-MAF-4* regulates *MAF* transcription by exploiting a chromosome loop with the promoter of *MAF* and its expression shift Th cells differentiation alternately toward Th1 or Th2 subtype (35). c-MAF was also identified in the complex network of TFs regulating Th17 cells as fundamental for the development of memory Th17 cells (75). HSF4 is one of the heat shock transcription factors that are involved in the suppressive function and cytokine production of Treg cells (76).

Concerning Treg cells, the comparison with literature highlighted some known Treg specific TFs, such as *FOXP3, IKZF2*, and *IKZF4*. *FOXP3* is the master regulator of Treg cell identity and regulates Ikaros family members, such as *IKZF2-4* (77), characterized as DNA binding proteins containing two zinc finger N-terminal domains (highly conserved) and protein binding domain (C-terminal). IKZF2, called Helios, is highly expressed in Treg cells and, by binding at its promoter, upregulates FoxP3 expression. Recently, lower Helios expression was detected in Treg cells from clinically isolated syndrome patients suggesting a less regulatory function (78). IKZF4, called Eos, facilitates FOXP3-mediated gene silencing in Treg cells (79).

Genes that are associated with Treg-CSRs belonged to GO categories related with chromatin remodeling and metabolic processes. The link between core regulatory regions with cluster of genes, that control cell metabolism, open a suggestive view of Treg plasticity dependent on metabolic shift. Indeed, Treg cells exhibit unique metabolic activities, characterized by low to modest glycolysis and elevated mechanistic target of rapamycin activity and nutrient metabolism, and these Treg-intrinsic metabolic pathways program Treg generation and activity. Treg cells have their own signaling and metabolic “preferences” that can drive and dictate their function and stability (80). Even more interestingly, genes associated with Th17-CSRs belonged to GO categories related with regulation and activation of immune response. This result perfectly matches with Th17 specific functions.

Here we focused our attention on CSRs associated with *RORC* and *FOXP3*, lineage- determining transcription factors that play a critical role in Th17 and Treg cell fate. Selected *RORC* associated CSRs included its promoter and an associated enhancer. *RORC* promoter was included in our analysis because of its biological relevance in transcriptional regulation of *RORC*. *FOXP3*-CSRs, instead, partially overlapped with conserved non-coding sequences (CNS). *Foxp3*-CNSs are three intronic enhancers identified at *Foxp3* gene locus, important for Treg cell. Epigenetic modifications at these regulatory regions are associated with Treg differentiation and functions (47). We found that *RORC*-CSRs and *FOXP3*-CSRs are ERα target in human PBMCs under Th17 polarization conditions and in PBMCs, Treg, and Th17 from MS patients. During Th17 polarization in presence of E2, we observed the enrichment of ERα binding at *FOXP3*-CSRs and at *RORC*-CSRs; these data go along with the enrichment of active marks at *FOXP3*-CSRs and repressive marks at *RORC* promoter, and with the enhanced *FOXP3* and reduced *RORC* expression. These epigenetic changes overlap with those that occur in PBMCs, and in purified Th17 and Treg cells during T3 in MS patients, where E2 reaches the highest levels. Altogether, these data suggest that ERα may induce chromatin remodeling by acting in opposite manners at two different loci (81). This effect could be ascribed to the recruitment of the different proteins in the regulatory complexes that may involve ERα as a key player for the switch between Th17 and Treg cells (23). It is plausible that during pregnancy, the gradual and continuous exposure to high levels of estrogen, can act both in the prevention of differentiation or in transdifferentiation processes.

Of particular interest was the observation that epigenetic modifications on RORC and FOXP3 loci occurs, not only at their promoters, but also at their enhancers. Studies aimed at the pharmacological targeting of epigenetic mechanisms made the exciting observation that SEs are particularly vulnerable to various inhibitors of transcriptional activation (82–84). Indeed, treating human CD4+ T cells from healthy controls with the JAK inhibitor tofacitinib selectively targeted rheumatoid arthritis risk genes controlled by SEs (85), while exposure of CD4+ T cells from Juvenile idiopathic arthritis (JIA) patients to the BET protein inhibitor JQ1 preferentially inhibited JIA-specific super-enhancer driven gene expression. BET protein inhibition was also shown to selectively block human Th17 differentiation and protect mice from experimentally induced autoimmunity (86). The identified SE at RORC and FOXP3 together with other TF identified in our analysis need to be deeply investigated and could be used, in the future, as “epigenetic drugs” for MS disease.

Important limitations of this study include the small amount of Th17 and Treg cell samples derived from MS patients during pregnancy. Our results show that Th17 cells percentage during pregnancy is reduced respect to MS active state and this is a peculiar feature of MS disease because higher Th17 cells levels mark the pathological condition and are instead absent in healthy donor, as we previously showed (3). Treg cells percentage increased in both MS patients and healthy donors during pregnancy, respect to non-pregnant state. Our results were expected for flow cytometry analysis, in addition we performed histone marks analysis at the FOXP3 genomic regulatory regions, partially overlapped with CNS regions associated with autoimmunity (47). Treg cell features in MS patients are associated with proliferation rate and cytokines expression dysregulation, and these alterations can emerge during pregnancy (87–89). We found that epigenetic modifications in pregnancy changed between T3 respect to PP in MS patients, but not in healthy donors. These results not completely explain the difference observed for Th17 and Treg cell levels. To address this point further analyses are necessary; single cell data- sequencing approach could reveal pathological state features linking surface- antigen makers with genomic, epigenetic and gene expression profiles.

In summary, here, we show that Th17 and Treg cells from pregnant MS patients have a peculiar epigenetic profile that could be associated with ERα-mediated estrogen effects. Pregnancy and autoimmunity are, indeed, challenging situations for the immune system. Treg and Th17 cells play a dominant role in both, although with opposing profiles: Treg cells activation ensures pregnancy success; in parallel, Th17 cells are important players in the development and progression of autoimmune diseases such as MS. Therefore, pregnancy condition mimics the pathological change of the balance between Treg and Th17 cells that occurs during relapsing- remitting disease course. This study offers an initial molecular understanding of the regulatory mechanisms ensuing during pregnancy and the identified CSRs may represent potential biomarkers for monitoring disease activity and progression or new potential therapeutic targets.

## Ethics Statement

Comitato etico interaziendale A.O.U. San Luigi Gonzaga. Prof. Francesco Di Carlo (presidente). Studio osservazionale Identificazione di nuovi marcatori epigenetici per seguire il decorso della sclerosi multipla.

## Author Contributions

MC and SaC designed the study and interpreted the data. AI, AlM, and MD performed and analyzed molecular biology experiments. SR, VB, II, and FN performed and analyzed immunological experiments. AlM, GF, and FC performed bioinformatic analysis. MC, SD, and LD, Torino/Brescia/Napoli groups provided patients samples, collected and analyzed the clinical data. AI, SR, and AV contributed to write and revise the manuscript, SaC and MC wrote the manuscript. MC, SD, LD, SiC, TT, MT, CC, GP, AnM, RL, VBM, and LD, Torino/Brescia/Napoli groups provided patients samples, collected and analyzed the clinical data.

### Conflict of Interest Statement

AV is employed by Merck Serono S.p.A. The remaining authors declare that the research was conducted in the absence of any commercial or financial relationships that could be construed as a potential conflict of interest.
