# Supplementary material for: Pregnancy Epigenetic Signature in T Helper 17 and T Regulatory Cells in Multiple Sclerosis
Source: Front Immunol. 2019 Jan 8;9:3075. doi: 10.3389/fimmu.2018.03075 (PMC6331474; doi:10.3389/fimmu.2018.03075)
Supplement: Supplementary file 3 [file Data_Sheet_3.docx]

| **Name** | **Amplified genomic region** | **Sequence** |
| --- | --- | --- |
|  |  |  |
| FOXP3 promoter fw | >chrX:49122053+49122218 166bp | TAATGCATCCATCCTCACGA |
| FOXP3 promoter rv |  | ATGATGGCGGATATTTGGAA |
| RORC promoter fw | >chr1:151804768-151804878 111bp | CAAGAGCAGCAAGGGTTAGG |
| RORC promoter rv |  | TTGGGGGACTGTGTCTCTTC |
| FOXP3 enhancer fw | [>chrX:49124092-49124276 185bp](https://genome.ucsc.edu/cgi-bin/hgTracks?hgsid=482041653_p5dgteSMeUf2OoUpFxW1dqMKc9Xp&db=hg19&position=chrX:49124092-49124276&hgPcrResult=pack) | TCCACATTCAGGCCCTAGAC |
| FOXP3 enhancer rv |  | AAGGGGACTCGGAGGTTAGA |
| FOXP3 intronic enhancer fw | [>chrX:49116479-49116672 194bp](https://genome.ucsc.edu/cgi-bin/hgTracks?hgsid=482041653_p5dgteSMeUf2OoUpFxW1dqMKc9Xp&db=hg19&position=chrX:49116479-49116672&hgPcrResult=pack) | TTCTTGGCTTGAAGGAGCAT |
| FOXP3 intronic enhancer rv |  | TGCTGAGGGAGATGAGTGTG |
| RORC enhancer fw | [>chr1:151821847-151821946 100bp](https://genome.ucsc.edu/cgi-bin/hgTracks?hgsid=482041653_p5dgteSMeUf2OoUpFxW1dqMKc9Xp&db=hg19&position=chr1:151821847-151821946&hgPcrResult=pack) | GAGGGAAGGGTCTGGAAAAG |
| RORC enhancer rv |  | ACAGCCCATTCTCCACAGAG |

**Table S3. Primers designed for ChIP followed by qPCR experiments on FOXP3 and RORC CSRs.**
