# Supplementary material for: Pregnancy Epigenetic Signature in T Helper 17 and T Regulatory Cells in Multiple Sclerosis
Source: Front Immunol. 2019 Jan 8;9:3075. doi: 10.3389/fimmu.2018.03075 (PMC6331474; doi:10.3389/fimmu.2018.03075)
Supplement: Supplementary file 4 [file Image_1.pdf]

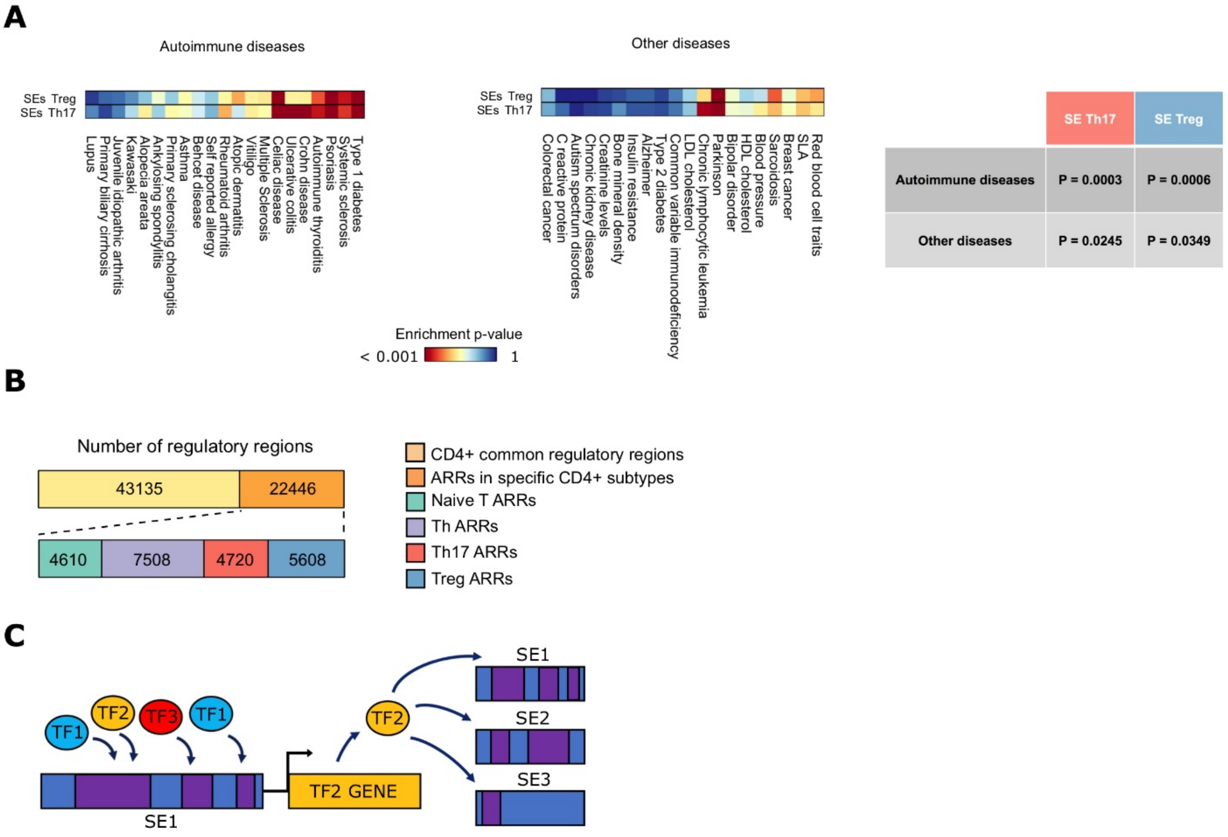

Fig.S1. Supplementary bioinformatics results. A. Results of SNPs analysis performed on predicted SEs in Th17 and Treg cells. Heatmap shows results of the single-trait-associated SNPs enrichment analysis. Table shows results of the enrichment analysis considering the two groups of diseases as a single trait. **B.** (RR) identified by considering a set of six regulatory states (emission parameter of H3K27ac and H3K4me1 ≥ 75) from CD4+ genome segmentation analysis (Roadmap Epigenomics Consortium, 2015).Comparison of RRs among CD4+ cells subtypes define active regulatory regions (ARR) in a specific CD4+ subtype. **C.** Methodology used for inferring Th17 and Treg networks.
